# Avoiding Punishment Is Its Own Reward

**DOI:** 10.1371/journal.pbio.0040247

**Published:** 2006-07-04

**Authors:** Liza Gross

For my now-departed, wonderful old cat named Bear, life didn't get any better than raw shrimp. Seeing the little white package emerge from the fridge always caught his attention, but what set him into high-shriek mode was the sound of shrimp being peeled under running water—he knew culinary bliss was at hand. Bear's behavior was perfectly in keeping with the theory of reinforcement learning: through instrumental conditioning, animals learn to choose responses associated with producing favorable outcomes and avoiding unpleasant ones—typically by learning to associate two normally unrelated stimuli. The shrimp reward reinforced associations between stimulus (the sound of peeling and washing, rather than the sight of shrimp) and response (expectant wailing).

The flipside of reward learning, avoidance learning, doesn't fit so neatly into the framework of reinforcement theories. Reinforcement theory predicts that behavior should rapidly disappear in the absence of explicit reinforcement. But studies show that once an animal manages to avoid punishment—for example, when a monkey learns to avoid a bitter drink by pressing a particular button—it may continue to perform the avoidance response even when it never experiences negative feedback again.

This apparent disconnect between avoidance learning and reinforcement theory could be resolved if avoiding punishment is itself a reward, a hypothesis that intrigued Hackjin Kim, Shinsuke Shimojo, and John O'Doherty. This possibility has been proposed before, but never tested. In a new study, Kim et al. investigated this question by scanning the brains of humans performing a simple instrumental conditioning task. A brain area called the medial orbitofrontal cortex (OFC) has been linked to reward-related stimuli, particularly when the reward involves money. The researchers reasoned that if avoidance learning and reward were equivalent, then the OFC should be activated in both contexts. If they are distinct cognitive processes, then each process should activate different regions.

Sixteen people participated in the study, during which they could either lose or win one dollar in an instrumental choice task. During the experimental trials, participants selected one of two fractal images presented on a screen. After a fractal was chosen, it became brighter, and four seconds later the participant got one of four types of feedback: reward (a picture of a dollar bill and the message, “You win $1!”), negative outcome (same image, with the text, “You lost $1!”), neutral (a scrambled bill with the text, “No change”), or nothing (a blank screen). During reward trials, the choice led to a high or low probability of reward (earning a dollar); during avoidance trials, the choice led to a high or low probability of avoiding a negative outcome (losing a dollar).

Over time, participants learned to choose fractals associated with a greater probability of reward and a lower probability of a negative outcome. And, as predicted, the medial OFC showed a higher response when participants chose an option that resulted in not losing the dollar or in winning it. Conversely, when participants' choices resulted in negative outcomes—and when there was no reward offered—OFC activity declined. Compared to neutral trials, reward and avoidance events produced significantly greater brain activity, while negative outcomes and neutral events linked to no chance of reward resulted in significantly decreased activity. Kim et al. argue that these functional magnetic resonance imaging (fMRI) results “provide direct evidence” that avoiding bad outcomes and receiving a reward provoke a similar response in the medial OFC.

The expectation of reward also produced heightened activity in the medial and lateral OFC. To analyze the learning response during reward and avoidance trials, the researchers input the results of the behavioral experiments into a computational reinforcement model. As participants received rewards over the course of learning, those choices resulting in reward increased in value; by contrast, the value of choices resulting in bad outcomes decreased. As links between actions and their outcomes become clearer, the wisdom or folly of a choice also becomes clearer.

Avoiding negative outcomes and receiving rewards amount to the same thing for the brain: achieving a goal. Reward serves as an external signal that reinforces behavior associated with a positive outcome, Kim et al. explain, and punishment amounts to an intrinsic reward signal that reinforces actions linked to avoiding bad outcomes. With fMRI evidence connecting avoidance and reward circuits, researchers can now determine which neuron populations within the OFC contribute to the avoidance–reward response—and perhaps shed light on the neurobiological roots of pathological risk-seeking behavior.

## 

**Figure:**
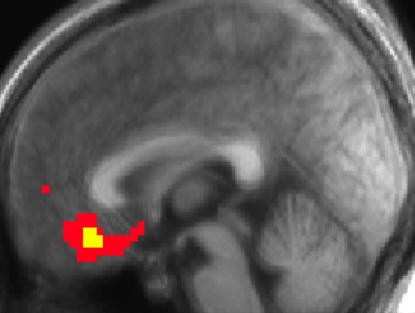
The same areas of the brain–in the medial orbitofrontal cortex–are engaged when people receive a reward or avoid a negative outcome.

